# Understanding the acetylome: translating targeted proteomics into meaningful physiology

**DOI:** 10.1152/ajpcell.00399.2013

**Published:** 2014-09-03

**Authors:** Andrew Philp, Thomas Rowland, Joaquin Perez-Schindler, Simon Schenk

**Affiliations:** ^1^School of Sport, Exercise, and Rehabilitation Sciences, University of Birmingham, Birmingham, United Kingdom; and; ^2^Department of Orthopaedic Surgery, University of California, San Diego, La Jolla, California

**Keywords:** mitochondria, muscle, adaptation, exercise, metabolism

## Abstract

It is well established that exercise elicits a finely tuned adaptive response in skeletal muscle, with contraction frequency, duration, and recovery shaping skeletal muscle plasticity. Given the power of physical activity to regulate metabolic health, numerous research groups have focused on the molecular mechanisms that sense, interpret, and translate this contractile signal into postexercise adaptation. While our current understanding is that contraction-sensitive allosteric factors (e.g., Ca^2+^, AMP, NAD^+^, and acetyl-CoA) initiate signaling changes, how the muscle translates changes in these factors into the appropriate adaptive response remains poorly understood. During the past decade, systems biology approaches, utilizing “omics” screening techniques, have allowed researchers to define global processes of regulation with incredible sensitivity and specificity. As a result, physiologists are now able to study substrate flux with stable isotope tracers in combination with metabolomic approaches and to coordinate these functional changes with proteomic and transcriptomic analysis. In this review, we highlight lysine acetylation as an important posttranslational modification in skeletal muscle. We discuss the evolution of acetylation research and detail how large proteomic screens in diverse metabolic systems have led to the current hypothesis that acetylation may be a fundamental mechanism to fine-tune metabolic adaptation in skeletal muscle.

posttranslational modification (PTM) of protein targets is a fundamental mechanism regulating cellular biological function. It is readily accepted that PTM is a pivotal process conveying environmental stresses to physiologically diverse processes such as cell cycle regulation, growth, autophagy, and apoptosis (7, 35, 79). While a considerable amount of research has focused on the PTM phosphorylation (35), recent technological advances have meant that it is now possible to study alternate PTMs, such as acetylation, methylation, and ubiquitination, on a global scale.

A role for acetylation in the regulation of gene transcription was first proposed by Allfrey et al. (5) following observations that RNA synthesis rates correlated with the acetylation status of core histone tails (Fig. 1). Indeed, subsequent analysis revealed that each of the four core histones (H2A, H2B, H3, and H4) within the nucleosome contained multiple lysine residues that undergo reversible acetylation (1, 32, 63, 76). These modifications also appeared functionally relevant, as an enrichment of acetylated histones was observed in transcribed DNA sequences (62). Acetylation results in neutralization of lysine residues located in amino-terminal domains of histones (62). Functionally, this is important, as histone tails protrude from the chromatin polymer and, as such, provide a platform for interacting and regulating proteins that remodel chromatin (33, 62). Acetylation-mediated change in the charge of histone tails is thought to weaken histone-DNA contact (5), modulate histone-histone interaction between adjacent nucleosomes (71), and affect interaction between histones and regulatory proteins (33). Collectively, these modifications result in altered structure and folding of nucleosomes, which collectively leads to a more open and permissive chromatin environment for transcriptional activation (62).

**Fig. 1.**
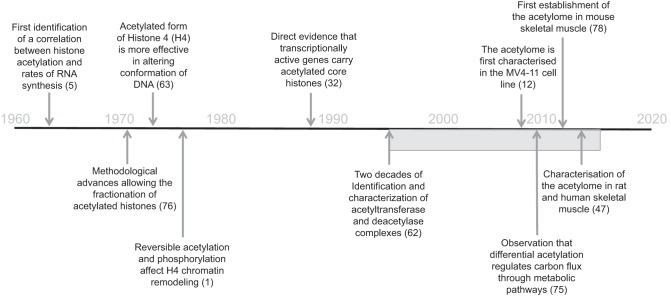
Timeline of key events leading to characterization of the acetylome in skeletal muscle.

## Characterization of the Acetylome

In contrast to gene regulation, the concept that reversible acetylation may affect the function of nonhistone substrates is a more recent discovery (45) (Fig. 1). The widespread nature of protein acetylation became strikingly apparent following the recent characterization of the “acetylome” by Matthias Mann's group (12). Utilizing acetylation-based immunoprecipitation to enrich acetylated peptides from MV4-11 cells (a human acute myeloid leukemia cell line), followed by high-resolution mass spectrometry (MS), Choudhary et al. identified ∼1,000 protein acetylation sites. However, this approach also appeared to present some limitations, with coverage apparently affected by a high background level of nonacetylated proteins. To circumvent this issue, Choudhary et al. examined overlapping signatures in two additional human cell lines (A549 and Jurkat cells), further enhanced the sensitivity of analysis by use of isoelectric focusing to separate acetylated peptides, and applied a stable isotope labeling of amino acids in cell culture (SILAC) approach to detect global acetylation changes following incubation with the histone deacetylase (HDAC) inhibitors suberoylanilide hydroxamic acid (SAHA) and MS-275. The net result of this approach was the identification of 3,600 lysine acetylation sites on 1,750 proteins (12). Interestingly, when Choudhary et al. examined the phylogenetic conservation of lysine-acetylated proteins compared with the proteome, lysine-acetylated proteins were significantly more conserved across the evolutionary tree, indicative of additional selective pressure. This level of conservation was comparable to phosphorylation, placing lysine acetylation as a broad and abundant PTM (57). Further evidence of the breadth of lysine acetylation was provided when acetylated proteins were assigned relative to gene ontology (GO) annotations (Table 1). A large proportion of acetylated proteins were enriched in mitochondrial and nuclear cellular compartments; in addition, >300 cytoplasmic proteins, representing ∼700 acetylation sites, were identified. Thus, acetylation appeared to modulate the protein/enzyme activity of numerous metabolic proteins, in addition to substrate transporters, receptors, and scaffolding proteins. Furthermore, the functional specificity of acetylation was highlighted, as identified acetylation sites were frequently located in regions with ordered secondary structure. When acetylation was compared with phosphorylation, which typically occurs in unstructured regions of proteins, such as hinges and loops (23, 35, 49), it appeared that not only was acetylation occurring in multisite regions in numerous proteins, but also that acetylation was targeting functionally important regions within these proteins. A final functional observation that warrants attention was that 14-3-3-binding proteins undergo multisite reversible acetylation (12). 14-3-3 proteins are widely expressed and function by binding phosphorylated proteins (phosphoserine and phosphothreonine) to facilitate their interaction with target substrates. To study the functional effect of 14-3-3 acetylation, Choudhary et al. mutated four lysine residues (K50, K69, K118, and K123) that are highly conserved among 14-3-3 proteins. By mutating the lysine residue to glutamine (K-to-Q), they could mimic acetylation, whereas a lysine-to-arginine substitution (K-to-R) prevented acetylation (12). Then they assessed the functional consequence of lysine mutation on 14-3-3-associated interaction with phosphopeptides. A triple-acetylation (K50 + K118 + K123Q) mutant did not bind phosphopeptides, indicating that acetylation directly affects the capacity of 14-3-3 proteins to interact with phosphorylated proteins (12). Thus these data demonstrate distinct cross talk between acetylation and phosphorylation and indicate that acetylation, in addition to direct modulation of cellular function, may also regulate phosphorylation signaling events through the 14-3-3 interactome.

**Table 1.** Characterization of acetylated proteins by function

Cellular Functional Categories and Protein Classes	No. of Acetylated Proteins	No. of Acetylation Sites
DNA replication	52	98
DNA damage and repair	72	167
Chromatin remodeling	26	46
Cell cycle	132	243
RNA transcription	31	71
RNA splicing	109	206
Nuclear hormone signaling	9	22
Nuclear transport	17	41
Cytoskeleton reorganization	50	137
Nucleotide exchange factors	55	92
Endocytosis and vesicular trafficking	39	62
DNA/RNA helicases	46	105
Ubiquitin ligases and deubiquitylases	46	70
Protein kinases	47	71
Acetyltransferases and deacetylases	21	61
Methyltransferases and demethylases	12	34
Transcription factors	29	40
Histones	15	61
Adaptor proteins	14	40
Chaperones	40	127
Ribosomal proteins	75	136

Following gene ontology annotation, acetylated proteins were characterized by function, with mass spectrometry analysis allowing identification of the number of acetylation sites on each target. [From Choudhary et al. (12).]

## Does Differential Acetylation Signatures Translate Into Functional Modifications?

Given the large number and the diverse cellular location of the acetylated proteins identified by Choudhary et al. (12), subsequent questions arose as to the role and importance of acetylation for regulating cellular function. These questions were subsequently addressed by Wang et al. (75), who studied acetylation in the prokaryote *Salmonella enterica*. This model is interesting, as unlike higher organisms, *S. enterica* contain only one major bacterial acetyltransferase (AT), Pat, and one nicotinamide adenine dinucleotide (NAD^+^)-dependent deacetylase (DAC), CobB, meaning that dramatic alteration of acetylation status in this model system via targeted disruption of Pat/CobB activity is relatively straightforward (75). To determine how lysine acetylation globally regulates metabolism, Wang et al. performed SILAC in *S. enterica* exposed to glucose, thereby directing the model to a glycolysis-dependent metabolic state, or to citrate, which induces oxidative/gluconeogenic metabolic preference. This approach identified 15 enzymes that had increased acetylation in the glucose condition compared with the citrate condition, directly linking carbon availability to altered acetylation (75). Functionally, this metabolic preference also paralleled growth rate, as glucose induction/acetylation resulted in increased growth, a condition that was mimicked via deletion or inhibition of CobB. To directly test the association between acetylation and metabolic flux, *S. enterica* were again grown in glucose/citrate conditions; however, carbon flux was directly measured using a [^13^C]glucose/citrate tracer followed by gas chromatography (GC)-MS analysis. This approach allowed Wang et al. to calculate carbon flux through glycolysis, gluconeogenesis, the tricarboxylic acid (TCA) cycle, and glyoxylate bypass. Furthermore, deletion mutants of Pat (ΔPat) and CobB (ΔCobB) then allowed the direct testing of the association between acetylation and metabolic flux. In glucose conditions, a significant elevation in glycolytic/gluconeogenic flux was observed in ΔCobB compared with wild-type/ΔPat. In contrast, after citrate induction, the ΔPat mutant demonstrated increased glyoxylate/TCA flux compared with wild-type/ΔCobB. Thus, glucose-mediated acetylation (via increased AT activity) resulted in increased glycolysis/gluconeogenesis, whereas citrate-mediated deacetylation (via increased DAC activity) resulted in increased glyoxylate/TCA flux (75). Finally, Wang et al. examined the direct effect of acetylation on the activity of three rate-limiting enzymes: glyceraldehyde phosphate dehydrogenase (GapA), which is involved in glycolysis/gluconeogenesis, and isocitrate lyase (AceA) and isocitrate dehydrogenase kinase/phosphatase (AceK), which are involved in the distribution of isocitrate into the TCA cycle and glyoxylate pathways when citrate is in abundance. Immunoprecipitation followed by pan-acetyl-lysine immunoblotting demonstrated that GapA, AceA, and AceK are hyperacetylated in the ΔCobB strain, concomitant with increased glycolytic flux, whereas differential deacetylation was observed in the ΔPat mutant. Further analysis directly demonstrated that metabolic flux profiles showed distinct patterns, depending on the substrate and level of acetylation. When the carbon source was citrate, metabolism bypassed glyoxylate and metabolism shifted to a gluconeogenic-predominant pathway in coordination with increased acetylation. Collectively, this work is fundamentally important, as it was the first direct evidence of the sensitivity of reversible acetylation to cellular substrate status and connected the activity of principal ATs/DACs to metabolic partitioning (75). Besides the action of enzymes such as Pat and CobB, global acetylation in *Escherichia coli* can also be regulated by the abundance of metabolites such as acetate, which is a precursor of acetyl-phosphate (77). Genetic deletion of the enzymes regulating acetyl-phosphate production (Pta) and removal (AckA) results in lower and higher global acetylation levels, respectively, in growth-arrested cells (77). Thus, this experimental model represents a condition in which protein acetylation is directly regulated by acetyl-phosphate in an enzyme-independent way, although the relevance of this mechanism in mammalian physiology remains to be determined.

The work from Choudhary et al. (12) and Wang et al. (75) was pioneering, as it utilized global MS analysis of acetylated peptides to characterize global alterations in lysine acetylation. Combining this approach with targeted inhibition (12) or genetic deletion (75) of ATs/DACs allowed the researchers to manipulate the acetylation signature to probe cellular function/interaction and the consequence of this adaptive response in metabolic regulation. However, as with any study utilizing reductionist approaches, it was still unclear whether acetylation could command such a dramatic level of regulation in higher organisms in which substrate partitioning has a vastly different complexity of regulation.

Zhao et al. (80) provided the first evidence that the aforementioned acetylome dataset (12, 75) may be translatable to higher organisms when they determined protein acetylomic signatures in human liver samples. After acetyl-lysine immunoprecipitation of nuclear, mitochondrial, and cytosolic fractions, tandem liquid chromatography (LC)-tandem MS (LC/LC-MS/MS) analysis identified 1,300 acetylated peptides, which matched 1,047 distinct human proteins (80). Interestingly, this approach provided substantially higher coverage than previous studies, which identified 195 acetylated proteins from a mouse liver preparation (40). Furthermore, only 240 of the proteins identified by Choudhary et al. (12) were identified by Zhao et al., indicating substantial variability between cell lines and intact tissue. It was striking that almost every enzyme involved in glycolysis, gluconeogenesis, the TCA cycle, the urea cycle, fatty acid metabolism, and glycogen metabolism were acetylated (80), providing direct evidence that acetylation is a prevalent PTM in vivo.

**Fig. 2.**
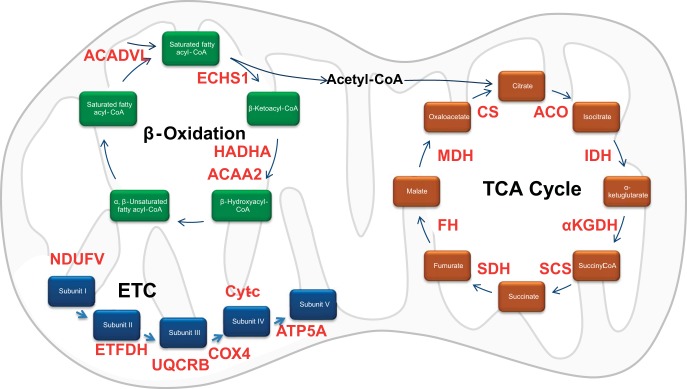
The majority of mitochondrial proteins/enzymes undergo reversible lysine acetylation. Each protein involved in β-oxidation, the tricarboxylic acid (TCA) cycle, and the electron transport chain (ETC) contain multiple acetylation residues in rodent and human skeletal muscle. Each metabolite of the respective mitochondrial pathway is identified in colored boxes; acetylated protein/enzyme regulating substrate oxidation is highlighted in red. ACADVL, acyl-CoA dehydrogenase, very long chain; ECHS1, enoyl-CoA dehydrogenase, short chain 1; HADHA, hydroxyacyl-CoA dehydrogenase; ACAA2, acetyl-CoA acyltransferase 2; NDUFV, NADH dehydrogenase (ubiquinone) flavoprotein; ETFDH, electron-transferring-flavoprotein dehydrogenase; UQCRB, ubiquinol-cytochrome *c* reductase-binding protein; COX4, cytochrome oxidase 4; Cytc, cytochrome *c*; ATP5A, ATP synthase subunit α; ACO, aconitase; IDH, isocitrate dehydrogenase; αKGDH, α-ketoglutarate dehydrogenase; SCS, succinyl-CoA synthase; SDH, succinate dehydrogenase; FH, fumarate hydratase; MDH, malate dehydrogenase; CS, citrate synthase. [Modified from data reported by Lundby et al. (47).]

The large proteomic datasets described above have provided an abundance of information relating to the coordinated interplay between acetylation and metabolism. However, translating, interpreting, and understanding this acetylation blueprint in vivo, within highly metabolic tissues such as skeletal muscle, has been more challenging (59). Initially, this limitation was addressed in part by Yang et al. (78), who determined the cytoplasmic/mitochondrial acetylome of different mouse tissues, including liver, heart, brain, and skeletal muscle, among others. Using LC-MS/MS, Yang et al. (78) found that acetylation of a subset of 31 proteins involved in energy metabolism was regulated in a tissue-specific manner following fasting/refeeding, and showed that in insulin-sensitive tissues (e.g., skeletal muscle), acetylation of these proteins is reduced by refeeding, suggesting a possible role in regulation of substrate utilization. Subsequently, Lundby et al. (47) reported a comprehensive characterization of the acetylome in rat and human skeletal muscle. Analysis of a tissue library of rat origin (liver, spleen, pancreas, muscle, skin, thymus, kidney, perirenal fat, brown fat, brain, intestine, heart, lung, stomach, testis fat, and testis) showed that these tissues each contained ∼1,000 acetylated proteins, a number that seemed to increase in relation to the metabolic capacity of the tissue. To further probe the underlying biology of tissue-specific acetylation signatures, Lundby et al. (47) performed GO and pathway (Reactome) analysis to show that acetylation patterns in skeletal muscle reflected major energy-consuming processes, with significant enrichment of proteins involved in muscle contraction and metabolic function. In fact, a staggering 80% of the proteins comprising the GO/pathway term “striated muscle contraction” were observed to be acetylated, with mitochondria demonstrating the greatest subcellular acetylation “pool” in both rodent and human skeletal muscle, with an average of 5.6 acetylation sites per protein identified (Fig. 2). Thus, tissue-specific acetylation signatures appear to regulate function-specific processes. By evaluating identified acetylated proteins by their GO annotations, Lundby et al. (47) showed that the majority of acetylated proteins reside in either the cytoplasm or the nucleus, each containing ∼30% of the acetylome pool. Mitochondria and the plasma membrane contain ∼15% of acetylated proteins, with the remainder (endoplasmic reticulum, Golgi apparatus, and extracellular space) each harboring ∼5% (47). Lundby et al. (47) then compared the protein distribution from their acetylome with that previously reported in the phosphoprotein atlas (36, 48). This analysis revealed that the subcellular distribution of acetylated proteins is markedly different from the distribution of phosphorylated proteins; for example, the abundance of mitochondrial acetylated proteins was threefold higher than that of phosphorylated proteins. In contrast, twice as many proteins in the plasma membrane compartment undergo phosphorylation as acetylation (47).

Given the extensive coverage observed in their dataset, Lundby et al. (47) were then able to examine whether common lysine acetylation sequence motifs occurred in tissue- or cell-specific compartments. By analyzing the 12 amino acid residues flanking the identified acetylated lysine residue, Lundby et al. (47) demonstrated that cytosolic acetylation favors lysine residues containing glutamate flanking the acetylation site, whereas mitochondrial proteins have a preference for negatively charged residues in the immediate vicinity of the acetylation site, with an additional selectivity for hydrophobic residues (V/I/L/F) two amino acids upstream of the acetylation site (+2 position). The most distinct sequence motif reported was for nuclear proteins, where there was a strong prevalence of glycine residues downstream of the acetylation site, in the −1 position, and proline residues in the +1 position. Interestingly, Lundby et al. (47) also reported that the sequence pattern of the transcription factor acetylation followed a trend (G-AcK-P) similar to that of nuclear proteins. Finally, this nuclear/transcription factor acetylation sequence pattern differed substantially from that previously reported for histone acetylation (79), indicating that not only was this pattern substrate-specific, but it could also differentiate between protein and chromatin targets in the nucleus (47).

## Lysine Acetylation Is an Intricate Process of Cellular Metabolic Regulation

**Fig. 3.**
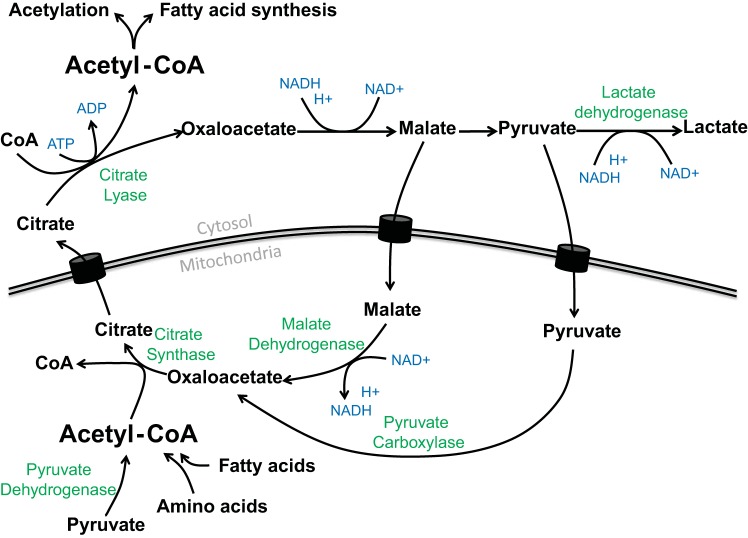
Metabolic pathways regulating acetyl-CoA content in skeletal muscle: processes of acetyl-CoA formation and utilization. Green text represents enzymes mediating each highlighted reaction; blue text represents nucleotide products of each enzymatic reaction.

Given the remarkable observations of these proteomic datasets, it clearly is now pertinent to understand, *1*) how lysine acetylation is regulated and controlled at the cellular level and *2*) what is the biological relevance of altered lysine acetylation with regard to metabolic health and disease? At any given time, the acetylation status of a protein is reflective of the balance between AT and DAC activity at target lysine residues. Importantly, acetylation status of both nuclear and mitochondrial proteins is also dependent on nonenzymatic factors that depend on fluctuations in metabolic flux and abundance of the acetyl donor acetyl-CoA (29, 72) (Fig. 3). Interestingly, Sutendra et al. (69) recently found that the pyruvate dehydrogenase complex regulates cell cycle progression via its translocation from the mitochondria to the nucleus, where it promotes acetyl-CoA synthesis and histone acetylation. Hence, protein acetylation and AT/DAC activity is, in turn, reflective of cellular energy status, as ATs utilize acetyl-CoA as a substrate, whereas class III HDACs are NAD^+^-dependent. Cellular acetyl-CoA and NAD^+^ pools are highly reflective of substrate flux through glucose, lipid, and ketogenic pathways (Fig. 3). As such, acetylation-deacetylation balance is highly reflective of cellular energy status, or, more precisely, substrate-mediated carbon flux, at any given time (25, 72). Accordingly, acetyl-CoA appears to promote an anabolic state in which it promotes mitochondrial protein acetylation and, consequently, decreases substrate oxidation, while nuclear acetyl-CoA produced by excessive energy supply or pyruvate dehydrogenase complex translocation to the nucleus seems to promote the expression of genes controlling energy storage and the cell cycle (29, 69, 72).

HDAC and nonhistone DAC, by definition, are classified as proteins with intrinsic enzymatic activity that are capable of removing acetyl groups from lysine residues. As their name suggests, HDACs were originally identified to perform this function on histone tails to modify chromatin (79). HDACs are categorized into five classes (I, IIa, IIb, III, and IV), consisting of two subfamilies. The first subfamily, known as the classical family, consists of the HDACs in classes I (HDAC1, 2, 3, and 8), IIa (HDAC4, 5, 7, and 9), IIb (HDAC6 and 10), and IV (HDAC11). The second family, known as the Sir2 family of NAD^+^-dependent enzymes, comprises class III [In-depth discussion of classical HDAC biology is beyond the scope of this review; for discussion of the regulation and biological roles of HDAC1-11 see recent comprehensive reviews (30, 44, 50)].

### Sirtuins: class III DACs.

The class III family of DACs, known as sirtuins (SIRTs), require NAD^+^ to catalyze the removal of an acetyl group from lysine residues, and also function as ADP-ribosyltransferases. Because of their reliance on NAD^+^ for their enzymatic activity, SIRTs are prime sensors of the metabolic state of the cell and have thus received much attention in the past decade (31). Seven mammalian SIRTs (SIRT1-7) have been identified. All SIRT proteins contain a conserved NAD^+^-binding site and catalytic domain, which has been termed the core domain (31). Phylogenic analyses of these domains have grouped SIRTs into four main classes (56): class I consists of SIRT1-3, class II of SIRT4, class III of SIRT5, and class IV of SIRT6-7 (56). SIRTs vary in their cellular localization, such that SIRT1 and 6 are predominantly localized to the nucleus, SIRT7 is nucleolar, SIRT3, 4, and 5 are primarily mitochondrial, and SIRT2 is predominantly cytoplasmic (31). These locations are, however, not strictly defined, and a SIRT's localization will likely depend on cell type, tissue, and environmental condition/stress (31, 41).

### SIRT1.

Considerable attention has been directed over the last decade toward elucidation of the role of SIRT proteins in numerous biological functions (24, 26, 31). Much of this work has utilized conditional SIRT mouse models and targeted deletion/overexpression in a variety of cell lines (31). In addition, more recent proteomic approaches have begun to explore the global effects of specific SIRT proteins. Chen et al. (11) recently characterized the SIRT1-dependent acetylome by combining SILAC approaches in SIRT1^+/+^ and SIRT1^−/−^ mouse embryonic fibroblasts with nano-HPLC/LTQ Orbitrap MS. Using this approach, they identified 4,623 nonredundant lysine acetylation sites in 1,800 proteins, with >94% of these sites conserved among mouse and human (11). This total eclipsed the 2,455 sites identified by Choudhary et al. (12), who used HDAC inhibitors to manipulate the acetylome, and reflects the strength of the SIRT1^+/+^ vs. SIRT1^−/−^ approach employed by Chen et al (11). Utilizing a protein-protein interaction network database (STRING) in combination with the MCODE detection algorithm, Chen et al. (11) identified 87 protein-protein interaction networks, including a ribosomal protein network, a mRNA splicing network, EGF stimulation networks, and a tRNA synthetase network. Hyperacetylation of proteins involved in DNA repair, the cell cycle, Notch signaling, and RNA splicing were overrepresented in SIRT1^−/−^ cells, indicating a direct role for SIRT1 in these processes, whereas knockout of SIRT1 had no effect on ribosome, proteasome, and glycolysis pathways (11). Performing a sequence motif analysis similar to that of Lundby et al. (47), Chen et al. (11) identified multiple motifs that are regulated by SIRT1. For example, motifs with a small nonpolar amino acid at the −1 or −2 position and a glutamic acid at the +2 position were significantly enriched in SIRT1^−/−^ cells (11). Finally, Chen et al. (11) provided an additional level of complexity in acetylation balance, as SIRT1 was shown to directly affect the activity of numerous AT protein complexes. Identified ATs that are SIRT1 substrates include cAMP response element-binding protein (CREB)-binding protein (CBP), Kat14 (Csrp2bp), Hat1, Kat5 [TAT 60-kDa interactive protein (TIP60)], Kat8 (Myst1), Kat7 (Myst2), Kat6a (Myst3), and Kat6b (Myst4) (11). When the previously described interaction between SIRT1 and 300-kDa protein (p300) is included (8), it is clear that, in addition to directly regulating protein acetylation, SIRT1 also indirectly influences global acetylation patterns by altering the biological activity of AT complexes (11).

### SIRT3: a mitochondrial-specific DAC.

While SIRT1 has been shown to target cytosolic and nuclear proteins, SIRT3 is classified as a mitochondrial-specific DAC. Therefore, given this cellular specificity, targeted proteomic approaches can allow the detailed examination of cellular organelle regulation. Evidence of the value of this approach was shown by Sol et al. (67), who performed SILAC labeling in SIRT3^−/−^ mouse embryonic fibroblasts (MEFs). Sol et al. (67) identified ∼900 acetylation sites across a number of key metabolic pathways. Hyperacetylation of specific proteins involved in long-chain lipid oxidation (very long-chain and long-chain acyl-CoA dehydrogenase), the TCA cycle (succinate dehydrogenase), and glycolysis/TCA cycle (pyruvate dehydrogenase) were observed in SIRT3^−/−^ MEFs, indicating direct regulation by SIRT3 (67). Deacetylation and activation of SIRT3 target proteins within mouse liver mitochondria have also been reported in response to the metabolic stress induced by acute fasting/refeeding and obesity (*ob/ob* mice), which seems to correlate with higher oxidative metabolism (68). In contrast, although genetic deletion of SIRT3 in conditional skeletal muscle or liver knockout mice increases mitochondrial protein acetylation, it does not affect tissue-specific mitochondrial function or whole body metabolism in animals fed chow or a high-fat diet (20). These data suggest that skeletal muscle and/or liver SIRT3 are not the main modulators of whole body metabolism, although SIRT3 might play a relevant role in different physiological/pathological contexts in which metabolic stress alters multiple signal pathways. Moreover, it seems that nonenzymatic acetylation modifies a broad spectrum of proteins within the mitochondria, suggesting that SIRT3 might regulate nonspecific mitochondrial protein acetylation, thereby fine-tuning the mitochondrial acetylome. Examination of this process in physiological contexts such as dietary restriction/overfeeding or following exercise would allow the precise role of SIRT3 in mitochondrial regulation to be fully elucidated.

## A Balanced Perspective: Defining Physiological Roles of ATs in Skeletal Muscle

As previously mentioned, the acetylation status of a protein at any given time is reflective of the combined action of a specific AT and a DAC, in addition to chemical acetylation due to alteration of mitochondrial matrix conditions. Therefore, it is surprising that research in the acetylation field is heavily weighted toward the study of DACs, rather than the study of ATs or understanding how substrate turnover within intracellular pools alters net acetylation balance. The pattern of this research has, in large part, been due to studies implying that SIRT1 is a longevity protein (24, 26) and showing that small-molecule activators of SIRT1 can be potent modulators of cellular metabolism (17, 54). Thus, momentum and significant pharmaceutical industry investment have promoted the view that ATs are less instrumental than HDAC and DACs in metabolic balance. However, without a balanced understanding of the biological processes that regulate chemical-, AT-, and DAC-mediated acetylation, interpretation of the physiological role of acetylation in the context of complex biological processes, such as aging and insulin resistance, will never truly be realized.

The first protein complex containing AT activity was identified >30 years ago (10). Since then, a number of ATs have been isolated from various organisms, with high conservation from yeast to humans (45). Furthermore, it is now recognized that ATs form multiple-subunit complexes, with the composition of these complexes fundamental in the biological activity and substrate specificity of the ATs contained within (45). Structural comparison of ATs has enabled their classification into distinct groups on the basis of their catalytic domains (45). General control of amino acid synthesis 5 (GCN5) is the founding member of the GCN5 *N*-acetyltransferase (GNAT) family, containing GCN5, p300/CBP-associated factor (PCAF), Elp3, Hat2, Hpa2, and Nut1; the MYST family contains Morf, Ybf2, Sas3, Sas2, and TIP60. Additional ATs of note are p300/CBP and Taf1 (45). Detailed coverage of each family, the interaction between complex proteins, and the functional characteristics of each AT complex are reported in detail by Lee and Workman (45).

## The GNAT Family: Dynamic Regulators of Metabolic Transcription

### GCN5 and PGC-1α.

GCN5 was initially identified as a transcriptional regulator in yeast (9, 62). It was later discovered that the yeast GCN5 protein had high sequence identity with p55, the catalytically active subunit of an A-type histone AT (HAT) isolated from *Tetrahymena* (9); this was an important finding, as it was one of the first observations linking transcriptional control with acetylation events (9). GCN5 is now well characterized to be the catalytically active AT of a number of HAT complexes in the GNAT family (45, 62). An important functional role for GCN5 in metabolic regulation was first uncovered by Lerin et al. (46) following the observation that GCN5 immunoprecipitated with the transcriptional coactivator peroxisome proliferator-activated receptor-γ coactivator 1α (PGC-1α). This observation was important, as tandem MS subsequently identified 13 acetylated lysine residues dispersed throughout PGC-1α that directly regulated PGC-1α activity (deacetylation increased PGC-1α transcriptional activity) (46). Lerin et al. (46) subsequently identified GCN5 to be the specific AT targeting PGC-1α in HEK 293 cells, with acetylation largely repressing PGC-1α transcriptional activity. Interestingly, Lerin et al. (46) showed that acetylation of PGC-1α leads to the sequestration of PGC-1α away from its target promoters and into nuclear foci.

Evidence in skeletal muscle came a few years later, when it was demonstrated that overexpression of GCN5 in C2C12 myotubes largely repressed PGC-1α-positive effects on mitochondrial and fatty acid oxidation enzyme gene expression (22). These effects were suggested to be due to increased GCN5 activity, given that SIRT1 inhibition with nicotinamide produced very similar results. Collectively, these series of studies suggested a model in which a low energy state is sensed by SIRT1, which subsequently functions to deacetylate and, thereby, activate PGC-1α transcriptional activity (14). On the other hand, in a high-energy state, acetylation of PGC-1α by GCN5 dominates and PGC-1α remains inactive and localized to nuclear foci (14).

Recently, we extended this work by demonstrating that GCN5 physically interacts with PGC-1α in skeletal muscle in vivo (58), with acute endurance exercise reducing the nuclear abundance of GCN5 in parallel to PGC-1α dissociation from GCN5 (58). This therefore led us to propose that exercise-induced changes in PGC-1α acetylation may be regulated by GCN5 (59). To our knowledge, the only upstream signal known to modify GCN5 is deacetylation by SIRT6, which functions to increase GCN5 activity and repression of PGC-1α (15). Interestingly, this study also identified two phosphorylation sites that may contribute to altered GCN5 activity (15); however, the physiological relevance of phosphorylation of GCN5 is unknown. The only other known regulation of GCN5 is at the transcriptional level, whereby the steroid receptor coactivator 3 can increase GCN5 gene expression (13). To our knowledge, however, there is no link between skeletal muscle contractile activity and steroid receptor coactivator 3 activity. However, given that exercise is known to activate several kinases, such as Ca^2+^-calmodulin protein kinase II, p38 mitogen-activated protein kinase, and AMP-activated protein kinase, it is tempting to speculate that GCN5 activity or localization may be affected by phosphorylation events triggered by contractile activity.

### GCN5 and PGC-1β.

In addition to the studies on PGC-1α, it has recently been shown that GCN5 also interacts with PGC-1β in HEK 293 cells, with ≥10 lysine acetylation residues identified (38). Using primary skeletal muscle myotubes, Kelly et al. (38) also demonstrated that GCN5 has a repressive effect on PGC-1β activity toward endogenous target genes. In addition, GCN5 repressed the stimulatory effect of PGC-1β on insulin-stimulated glucose uptake. Despite the fact that only one PGC-1β lysine residue is conserved with respect to PGC-1α, most of the acetylated residues were in homologous regions (38). Finally, a recent report showed that a GCN5 variant, GCN5L1, appears to be involved in acetylating mitochondrial proteins in vitro or in Hep G2 cells, thus acting against the known mitochondrial DAC SIRT3 (65). Interestingly, the acetylation changes induced by GCN5L1 only occur in the presence of mitochondrial extracts (65), suggesting that a multimeric complex is required for the effects of GCN5L1 (65). With this in mind and considering that the study did not utilize GCN5L1 mutants lacking AT activity, it is unclear whether GCN5L1 is specifically the AT mediating mitochondrial acetylation changes or part of a larger mitochondrial AT complex.

The studies described above demonstrate that GCN5 may play a central role in skeletal muscle physiology. However, this evidence is largely restricted to GCN5 action on PGC-1α/β. Thus there is an urgent need to characterize global GCN5 substrates if a clear picture of GCN5 function is to be formed. For physiologists interested in the functional role of GCN5 in skeletal muscle plasticity, an obvious experimental model is the use of transgenic mice. GCN5 knockout mice are, however, embryonically lethal (62); therefore, conditional knockout models are needed.

### PCAF.

The human PCAF protein is closely related to GCN5, with 73% sequence similarity (55). PCAF has been known for some time to be involved in muscle cell differentiation, following the observation that PCAF acetylates MyoD, thereby enhancing its DNA-binding stability (51). In addition, PCAF's interaction with MyoD allows recruitment to chromatin, where it may function to acetylate histones and activate transcription of muscle genes (51). Recently, it was demonstrated that inhibition of PCAF activity with the noncompetitive inhibitor embelin reduces myotube formation and PCAF-mediated MyoD acetylation. Furthermore, microarray analysis in C2C12 myoblasts undergoing differentiation indicates that PCAF may influence a number of myogenic genes beyond MyoD that collectively alter differentiation (51). With the exception of MyoD, little else is known about the nonhistone substrates of PCAF in adult skeletal muscle. It is of interest with regard to skeletal muscle plasticity that PCAF has been shown to associate with cardiomyocyte sarcomeres (27) and is implicated in acetylating the Z-disk protein muscle LIM protein (27), as well as myosin heavy chains (27).

### Summary.

Given the vast degree of acetylated proteins involved in skeletal muscle contraction (47), it will be interesting to see if future research identifies specific physiological functions for PCAF in skeletal muscle. As for GCN5 and GCN5L1, global determination of both nuclear and cytosolic targets of PCAF will uncover the nongenomic functions of these ATs, which represents a novel line of research. This approach might unmask new biological functions and even define the functional interplay between metabolic pathways and the regulation of gene expression mediated by these ATs. For instance, it seems likely that regulation of metabolic flux by GCN5L1-mediated mitochondrial protein acetylation might directly affect cytosolic levels of acetyl-CoA and gene expression.

## Is There a Potential Role of the MYST Family in Metabolic Regulation?

TIP60, as the name suggests, was originally identified via its interaction with the protein product of the TAT gene, which is found in the human immunodeficiency virus genome (62). It is a member of the MYST family of HATs, and the human TIP60 protein has been demonstrated to possess HAT activity (45). TIP60 was originally shown by Lerin et al. (46) to coimmunoprecipitate with PGC-1α; however, focus beyond this was centered on GCN5, once TIP60 was shown to be unable to acetylate PGC-1α in HEK 293 cells (46). Accordingly, Lerin et al. (46) suggested that the TIP60 complex might, instead, recruit PGC-1α to the promoters of genes involved in oxidative stress and DNA repair. Alternatively, perhaps TIP60 is recruited to DNA via its interaction with PGC-1α, where it functions to acetylate histones and, thereby, activate transcription of PGC-1α target genes.

With respect to skeletal muscle, it has recently been demonstrated that TIP60, in a similar manner to PCAF, interacts with MyoD to enhance its transcriptional activity and induce myoblast differentiation (39). Whether this was dependent on the AT activity of TIP60 was not answered. Nonetheless, there is low expression of TIP60 mRNA in adult murine skeletal muscle (45). It seems unlikely, therefore, that TIP60 plays a critical role in regulating differentiated adult skeletal muscle plasticity. It has, however, been shown that TIP60 may be an important part of the signal transduction events triggered by endothelins binding to their G protein-coupled receptors (39). This suggests a possible role in controlling vasoconstriction, which would, of course, affect nutrient and hormone delivery to skeletal muscle, particularly in response to the hyperemic effects of exercise. Further examination of the role of TIP60 is therefore clearly warranted.

## p300/CBP: Multifunctional Regulators of Cellular Metabolism

p300 and its homolog CBP are two closely related transcriptional coactivators that contain intrinsic AT activity (74). p300 was initially discovered due to its interaction with the E1A adenovirus (16), and CBP was discovered independently via its interaction with CREB (43). Not long thereafter, the two proteins were shown to contain high sequence homology (6) and, indeed, to share similar functions, although independent functions have begun to be discovered (45). While the role of p300/CBP in histone acetylation and transcriptional regulation is reasonably well characterized (62), relatively little is known about if and how p300/CBP may regulate skeletal muscle metabolism via nonhistone acetylation of protein targets. The possibility that p300 is a central regulator of skeletal muscle metabolism is quite high, given that isolated studies have identified a number of p300/CBP substrates with known important roles in skeletal muscle. These include, but are not limited to, PGC-1α (61, 73), myocyte enhancer factor 2 (MEF2) (81), MyoD (64), nuclear factor of activated T cells (NFATc1) (53), and S6 kinase 1 (S6K1) (18). With this in mind, it seems intuitive that p300/CBP would play a role in regulating skeletal muscle adaptation to exercise.

p300 and CBP have been shown to interact with PGC-1α (61, 73) and increase PGC-1α transcriptional activity. The stimulatory effect of p300/CBP on PGC-1α activity is, however, unlikely due to direct acetylation of PGC-1α (46) but may, instead, be due to histone acetylation at PGC-1α target genes (73). Beyond PGC-1α, a series of studies have demonstrated that p300 may be involved in fiber-type transitioning in skeletal muscle (52, 53). Interestingly, these series of studies built a pathway suggesting that increasing intracellular Ca^2+^ levels results in the translocation of cytoplasmic NFATc1 to the cell nucleus upon dephosphorylation by calcineurin, where it binds weakly to the myosin heavy chain β-subunit (MyHCβ) promoter. Concomitantly, increased Ca^2+^ also activates the MEK1-ERK1 pathway, which results in phosphorylation and activation of p300. In the nucleus, p300 acetylates NFATc1, thereby increasing its DNA-binding stability, and as a consequence, MyHCβ promoter activity is further increased (52, 53). Given that endurance exercise increases intracellular Ca^2+^ levels, it seems plausible that a similar pathway could regulate skeletal muscle plasticity. In addition, it has also been shown that CBP can acetylate a lysine residue on MEF2D, a site that can also undergo sumoylation (81). In this instance, it was suggested that acetylation functions to prevent the transcriptionally repressive modification of sumoylation (81). Interestingly, MEF2 is required to induce PGC-1α promoter activity in mouse skeletal muscle after contraction (2); it is also necessary for promoter activity of the GLUT4 gene (70) and plays a role in regulating oxidative capacity (60). Therefore, it could be speculated that p300/CBP-mediated acetylation of MEF2 may also be an important part of the acute postendurance exercise-signaling response that drives oxidative changes in response to endurance exercise.

Beyond oxidative metabolism, a growing number of reports link p300/CBP to the regulation of muscle mass. Indeed, p300 and CBP and their AT activity are required for phenylephrine-induced hypertrophy of cardiac cells (28). Interestingly, p300 has been shown to interact with S6K1 and S6K2 in vitro and upon serum stimulation in MCF-7 cells (19), with a specific lysine residue (K516) on S6K1 identified to be directly acetylated by p300 (18). Acetylation of S6K1 does not appear to be necessary for its activation (18) but may improve protein stability (18). This interaction has not been studied in skeletal muscle. In addition, p300 has also been implicated in various muscle-wasting conditions. Sepsis induction or dexamethasone treatment in rats leads to an increase in p300 expression, an increase in global AT activity, and a decrease in global DAC activity in extensor digitorum longus muscle (4). Therefore, it has been suggested that p300-driven hyperacetylation may regulate an increase in protein breakdown in sepsis (3). On the contrary, overexpression of p300 or CBP prevents an immobilization-induced increase in FOXO activity in rat soleus muscle, an effect dependent on AT activity (66). This suggests that acetylation may protect from immobilization-induced muscle wasting (66), which is contrary to the concept that hyperacetylation contributes to sepsis-induced muscle wasting (4). This disparity could be due to the different atrophy models used and/or muscle groups examined (4) but also suggests that additional research into the growth-promoting/atrophy-sparing effects of p300 is warranted.

It is important to note that although p300 and CBP share high sequence homology and interaction partners, studies that have investigated both p300 and CBP have noted distinct functions or substrate preferences (37). Among the studies described above that investigated both p300 and CBP, Senf et al. (66) reported that p300 and CBP have differential effects on FOXO1 and FOXO3a cellular localization, and Meissner et al. (53) noted that only p300 appears to interact with NFATc1. Thus it appears that the biological activities of p300 or CBP are not necessarily interchangeable and that it is important to investigate both of these ATs in isolation and in combination. From an integrative physiology perspective, this could be accomplished with single- and double-knockout animal models or AT-specific small-molecule inhibitors.

## The Next Challenge: Examination of Lysine Acetylation in a Physiological Context

The determination of the acetylome in diverse metabolic tissues now provides physiologists with a molecular blueprint to study the impact of this PTM in physiological and pathophysiological scenarios. Given that acetylation has been reported to be altered in aged (42) and insulin-resistant (34) skeletal muscle, this research promises to have substantial therapeutic relevance. Progress has already been substantially advanced by the use of conditional mouse models to study the role of HDAC and DAC proteins in cellular metabolic processes (21, 30). The logical step now is for complementary work to be performed in conditional AT mouse models with the use of AT-specific inhibitors/activators. Beyond this, translational studies in humans should be achievable, given the sensitivity of acetylation to substrate flux (72). Nutritional approaches to manipulate glucose and lipid metabolism, thereby altering NAD^+^ and acetyl-CoA availability, will impact AT and sirtuin activity, as will cellular stressors such as exercise and calorie restriction. It has been established that many of these interventions dramatically alter physiological responses; the question now is whether the underlying mechanism for this adaptation is a consequence of altered acetylation. By developing interaction between basic physiology and targeted “omic” approaches, we anticipate that future research will lead to the extension of the acetylome into meaningful physiological scenarios, which ultimately will uncover many fundamental levels of metabolic regulation in skeletal muscle.

## GRANTS

This publication was supported in part by National Institutes of Health Grants R01 AG-043120, R24 HD-050837, and P30 AR-058878 (to S. Schenk) and a Sir Henry Wellcome Postdoctoral Fellowship (to J. Perez-Schindler).

## DISCLOSURES

No conflicts of interest, financial or otherwise, are declared by the authors.

## AUTHOR CONTRIBUTIONS

A.P. prepared the figures; A.P., T.R., J.P.-S., and S.S. drafted the manuscript; A.P., T.R., J.P.-S., and S.S. edited and revised the manuscript; A.P., T.R., J.P.-S., and S.S. approved the final version of the manuscript.

## References

[1] Adler AJ Fasman GD Wangh LJ Allfrey VG. Altered conformational effects of naturally acetylated histone f2al (IV) in f2al-deoxyribonucleic acid complexes. Circular dichroism studies. J Biol Chem 249: 2911–2914, 1974 4828328

[2] Akimoto T Pohnert SC Li P Zhang M Gumbs C Rosenberg PB Williams RS Yan Z. Exercise stimulates Pgc-1α transcription in skeletal muscle through activation of the p38 MAPK pathway. J Biol Chem 280: 19587–19593, 2005 15767263 10.1074/jbc.M408862200

[3] Alamdari N Aversa Z Castillero E Hasselgren PO. Acetylation and deacetylation—novel factors in muscle wasting. Metab Clin Exp 62: 1–11, 2013 22626763 10.1016/j.metabol.2012.03.019 PMC3430797

[4] Alamdari N Smith IJ Aversa Z Hasselgren PO. Sepsis and glucocorticoids upregulate p300 and downregulate HDAC6 expression and activity in skeletal muscle. Am J Physiol Regul Integr Comp Physiol 299: R509–R520, 2010 20538901 10.1152/ajpregu.00858.2009 PMC2928620

[5] Allfrey VG Faulkner R Mirsky AE. Acetylation and methylation of histones and their possible role in the regulation of RNA synthesis. Proc Natl Acad Sci USA 51: 786–794, 1964 14172992 10.1073/pnas.51.5.786 PMC300163

[6] Arany Z Sellers WR Livingston DM Eckner R. E1A-associated p300 and CREB-associated CBP belong to a conserved family of coactivators. Cell 77: 799–800, 1994 8004670 10.1016/0092-8674(94)90127-9

[7] Bedford MT Clarke SG. Protein arginine methylation in mammals: who, what, and why. Mol Cell 33: 1–13, 2009 19150423 10.1016/j.molcel.2008.12.013 PMC3372459

[8] Bouras T Fu M Sauve AA Wang F Quong AA Perkins ND Hay RT Gu W Pestell RG. SIRT1 deacetylation and repression of p300 involves lysine residues 1020/1024 within the cell cycle regulatory domain 1. J Biol Chem 280: 10264–10276, 2005 15632193 10.1074/jbc.M408748200

[9] Brownell JE Zhou J Ranalli T Kobayashi R Edmondson DG Roth SY Allis CD. *Tetrahymena* histone acetyltransferase A: a homolog to yeast Gcn5p linking histone acetylation to gene activation. Cell 84: 843–851, 1996 8601308 10.1016/s0092-8674(00)81063-6

[10] Cano A Pestana A. Purification and properties of a histone acetyltransferase from *Artemia salina*, highly efficient with H1 histone. Eur J Biochem 97: 65–72, 1979 477673 10.1111/j.1432-1033.1979.tb13086.x

[11] Chen Y Zhao W Yang JS Cheng Z Luo H Lu Z Tan M Gu W Zhao Y. Quantitative acetylome analysis reveals the roles of SIRT1 in regulating diverse substrates and cellular pathways. Mol Cell Proteomics 11: 1048–1062, 2012 22826441 10.1074/mcp.M112.019547 PMC3494151

[12] Choudhary C Kumar C Gnad F Nielsen ML Rehman M Walther TC Olsen JV Mann M. Lysine acetylation targets protein complexes and co-regulates major cellular functions. Science 325: 834–840, 2009 19608861 10.1126/science.1175371

[13] Coste A Louet JF Lagouge M Lerin C Antal MC Meziane H Schoonjans K Puigserver P O'Malley BW Auwerx J. The genetic ablation of SRC-3 protects against obesity and improves insulin sensitivity by reducing the acetylation of PGC-1α. Proc Natl Acad Sci USA 105: 17187–17192, 2008 18957541 10.1073/pnas.0808207105 PMC2579399

[14] Dominy JE Jr. Lee Y Gerhart-Hines Z Puigserver P. Nutrient-dependent regulation of PGC-1α's acetylation state and metabolic function through the enzymatic activities of Sirt1/GCN5. Biochim Biophys Acta 1804: 1676–1683, 2010 20005308 10.1016/j.bbapap.2009.11.023 PMC2886158

[15] Dominy JE Jr. Lee Y Jedrychowski MP Chim H Jurczak MJ Camporez JP Ruan HB Feldman J Pierce K Mostoslavsky R Denu JM Clish CB Yang X Shulman GI Gygi SP Puigserver P. The deacetylase Sirt6 activates the acetyltransferase GCN5 and suppresses hepatic gluconeogenesis. Mol Cell 48: 900–913, 2012 23142079 10.1016/j.molcel.2012.09.030 PMC3534905

[16] Eckner R Ewen ME Newsome D Gerdes M DeCaprio JA Lawrence JB Livingston DM. Molecular cloning and functional analysis of the adenovirus E1A-associated 300-kD protein (p300) reveals a protein with properties of a transcriptional adaptor. Genes Dev 8: 869–884, 1994 7523245 10.1101/gad.8.8.869

[17] Feige JN Lagouge M Canto C Strehle A Houten SM Milne JC Lambert PD Mataki C Elliott PJ Auwerx J. Specific SIRT1 activation mimics low energy levels and protects against diet-induced metabolic disorders by enhancing fat oxidation. Cell Metab 8: 347–358, 2008 19046567 10.1016/j.cmet.2008.08.017

[18] Fenton TR Gwalter J Cramer R Gout IT. S6K1 is acetylated at lysine 516 in response to growth factor stimulation. Biochem Biophys Res Commun 398: 400–405, 2010 20599721 10.1016/j.bbrc.2010.06.081

[19] Fenton TR Gwalter J Ericsson J Gout IT. Histone acetyltransferases interact with and acetylate p70 ribosomal S6 kinases in vitro and in vivo. Int J Biochem Cell Biol 42: 359–366, 2010 19961954 10.1016/j.biocel.2009.11.022

[20] Fernandez-Marcos PJ Jeninga EH Canto C Harach T de Boer VC Andreux P Moullan N Pirinen E Yamamoto H Houten SM Schoonjans K Auwerx J. Muscle or liver-specific Sirt3 deficiency induces hyperacetylation of mitochondrial proteins without affecting global metabolic homeostasis. Sci Rep 2: 425, 2012 22645641 10.1038/srep00425 PMC3361023

[21] Finkel T Deng CX Mostoslavsky R. Recent progress in the biology and physiology of sirtuins. Nature 460: 587–591, 2009 19641587 10.1038/nature08197 PMC3727385

[22] Gerhart-Hines Z Rodgers JT Bare O Lerin C Kim SH Mostoslavsky R Alt FW Wu Z Puigserver P. Metabolic control of muscle mitochondrial function and fatty acid oxidation through SIRT1/PGC-1α. EMBO J 26: 1913–1923, 2007 17347648 10.1038/sj.emboj.7601633 PMC1847661

[23] Gnad F Ren S Cox J Olsen JV Macek B Oroshi M Mann M. PHOSIDA (phosphorylation site database): management, structural and evolutionary investigation, and prediction of phosphosites. Genome Biol 8: R250, 2007 18039369 10.1186/gb-2007-8-11-r250 PMC2258193

[24] Guarente L. Franklin H. Epstein lecture: Sirtuins, aging, and medicine. N Engl J Med 364: 2235–2244, 2011 21651395 10.1056/NEJMra1100831

[25] Guarente L. The logic linking protein acetylation and metabolism. Cell Metab 14: 151–153, 2011 21803285 10.1016/j.cmet.2011.07.007

[26] Guarente L. Sirtuins as potential targets for metabolic syndrome. Nature 444: 868–874, 2006 17167475 10.1038/nature05486

[27] Gupta MP Samant SA Smith SH Shroff SG. HDAC4 and PCAF bind to cardiac sarcomeres and play a role in regulating myofilament contractile activity. J Biol Chem 283: 10135–10146, 2008 18250163 10.1074/jbc.M710277200 PMC2442284

[28] Gusterson RJ Jazrawi E Adcock IM Latchman DS. The transcriptional co-activators CREB-binding protein (CBP) and p300 play a critical role in cardiac hypertrophy that is dependent on their histone acetyltransferase activity. J Biol Chem 278: 6838–6847, 2003 12477714 10.1074/jbc.M211762200

[29] Gut P Verdin E. The nexus of chromatin regulation and intermediary metabolism. Nature 502: 489–498, 2013 24153302 10.1038/nature12752

[30] Haberland M Montgomery RL Olson EN. The many roles of histone deacetylases in development and physiology: implications for disease and therapy. Nat Rev Genet 10: 32–42, 2009 19065135 10.1038/nrg2485 PMC3215088

[31] Haigis MC Sinclair DA. Mammalian sirtuins: biological insights and disease relevance. Annu Rev Pathol 5: 253–295, 2010 20078221 10.1146/annurev.pathol.4.110807.092250 PMC2866163

[32] Hebbes TR Thorne AW Crane-Robinson C. A direct link between core histone acetylation and transcriptionally active chromatin. EMBO J 7: 1395–1402, 1988 3409869 10.1002/j.1460-2075.1988.tb02956.x PMC458389

[33] Hecht A Laroche T Strahl-Bolsinger S Gasser SM Grunstein M. Histone H3 and H4 N-termini interact with SIR3 and SIR4 proteins: a molecular model for the formation of heterochromatin in yeast. Cell 80: 583–592, 1995 7867066 10.1016/0092-8674(95)90512-x

[34] Hirschey MD Shimazu T Jing E Grueter CA Collins AM Aouizerat B Stancakova A Goetzman E Lam MM Schwer B Stevens RD Muehlbauer MJ Kakar S Bass NM Kuusisto J Laakso M Alt FW Newgard CB Farese RV Jr. Kahn CR Verdin E. SIRT3 deficiency and mitochondrial protein hyperacetylation accelerate the development of the metabolic syndrome. Mol Cell 44: 177–190, 2011 21856199 10.1016/j.molcel.2011.07.019 PMC3563434

[35] Hunter T. The age of crosstalk: phosphorylation, ubiquitination, and beyond. Mol Cell 28: 730–738, 2007 18082598 10.1016/j.molcel.2007.11.019

[36] Huttlin EL Jedrychowski MP Elias JE Goswami T Rad R Beausoleil SA Villen J Haas W Sowa ME Gygi SP. A tissue-specific atlas of mouse protein phosphorylation and expression. Cell 143: 1174–1189, 2010 21183079 10.1016/j.cell.2010.12.001 PMC3035969

[37] Kalkhoven E. CBP and p300: HATs for different occasions. Biochem Pharmacol 68: 1145–1155, 2004 15313412 10.1016/j.bcp.2004.03.045

[38] Kelly TJ Lerin C Haas W Gygi SP Puigserver P. GCN5-mediated transcriptional control of the metabolic coactivator PGC-1β through lysine acetylation. J Biol Chem 284: 19945–19952, 2009 19491097 10.1074/jbc.M109.015164 PMC2740420

[39] Kim JW Jang SM Kim CH An JH Kang EJ Choi KH. Tip60 regulates myoblast differentiation by enhancing the transcriptional activity of MyoD via their physical interactions. FEBS J 278: 4394–4404, 2011 21936881 10.1111/j.1742-4658.2011.08362.x

[40] Kim SC Sprung R Chen Y Xu Y Ball H Pei J Cheng T Kho Y Xiao H Xiao L Grishin NV White M Yang XJ Zhao Y. Substrate and functional diversity of lysine acetylation revealed by a proteomics survey. Mol Cell 23: 607–618, 2006 16916647 10.1016/j.molcel.2006.06.026

[41] Kiran S Chatterjee N Singh S Kaul SC Wadhwa R Ramakrishna G. Intracellular distribution of human SIRT7 and mapping of the nuclear/nucleolar localization signal. FEBS J 280: 3451–3466, 2013 23680022 10.1111/febs.12346

[42] Koltai E Szabo Z Atalay M Boldogh I Naito H Goto S Nyakas C Radak Z. Exercise alters SIRT1, SIRT6, NAD and NAMPT levels in skeletal muscle of aged rats. Mech Ageing Dev 131: 21–28, 2010 19913571 10.1016/j.mad.2009.11.002 PMC2872991

[43] Kwok RP Lundblad JR Chrivia JC Richards JP Bachinger HP Brennan RG Roberts SG Green MR Goodman RH. Nuclear protein CBP is a coactivator for the transcription factor CREB. Nature 370: 223–226, 1994 7913207 10.1038/370223a0

[44] Lahue RS Frizzell A. Histone deacetylase complexes as caretakers of genome stability. Epigenetics 7: 806–810, 2012 22722985 10.4161/epi.20922 PMC3427275

[45] Lee KK Workman JL. Histone acetyltransferase complexes: one size doesn't fit all. Nat Rev Mol Cell Biol 8: 284–295, 2007 17380162 10.1038/nrm2145

[46] Lerin C Rodgers JT Kalume DE Kim SH Pandey A Puigserver P. GCN5 acetyltransferase complex controls glucose metabolism through transcriptional repression of PGC-1α. Cell Metab 3: 429–438, 2006 16753578 10.1016/j.cmet.2006.04.013

[47] Lundby A Lage K Weinert BT Bekker-Jensen DB Secher A Skovgaard T Kelstrup CD Dmytriyev A Choudhary C Lundby C Olsen JV. Proteomic analysis of lysine acetylation sites in rat tissues reveals organ specificity and subcellular patterns. Cell Rep 2: 419–431, 2012 22902405 10.1016/j.celrep.2012.07.006 PMC4103158

[48] Lundby A Secher A Lage K Nordsborg NB Dmytriyev A Lundby C Olsen JV. Quantitative maps of protein phosphorylation sites across 14 different rat organs and tissues. Nat Commun 3: 876, 2012 22673903 10.1038/ncomms1871 PMC3621391

[49] Malik R Nigg EA Korner R. Comparative conservation analysis of the human mitotic phosphoproteome. Bioinformatics 24: 1426–1432, 2008 18426804 10.1093/bioinformatics/btn197

[50] McGee SL Hargreaves M. Histone modifications and exercise adaptations. J Appl Physiol 110: 258–263, 2011 21030677 10.1152/japplphysiol.00979.2010

[51] McKinsey TA Zhang CL Olson EN. Control of muscle development by dueling HATs and HDACs. Curr Opin Genet Dev 11: 497–504, 2001 11532390 10.1016/s0959-437x(00)00224-0

[52] Meissner JD Freund R Krone D Umeda PK Chang KC Gros G Scheibe RJ. Extracellular signal-regulated kinase 1/2-mediated phosphorylation of p300 enhances myosin heavy chain I/β gene expression via acetylation of nuclear factor of activated T cells c1. Nucleic Acids Res 39: 5907–5925, 2011 21498542 10.1093/nar/gkr162 PMC3152325

[53] Meissner JD Umeda PK Chang KC Gros G Scheibe RJ. Activation of the β-myosin heavy chain promoter by MEF-2D, MyoD, p300, and the calcineurin/NFATc1 pathway. J Cell Physiol 211: 138–148, 2007 17111365 10.1002/jcp.20916

[54] Milne JC Lambert PD Schenk S Carney DP Smith JJ Gagne DJ Jin L Boss O Perni RB Vu CB Bemis JE Xie R Disch JS Ng PY Nunes JJ Lynch AV Yang H Galonek H Israelian K Choy W Iffland A Lavu S Medvedik O Sinclair DA Olefsky JM Jirousek MR Elliott PJ Westphal CH. Small molecule activators of SIRT1 as therapeutics for the treatment of type 2 diabetes. Nature 450: 712–716, 2007 18046409 10.1038/nature06261 PMC2753457

[55] Nagy Z Tora L. Distinct GCN5/PCAF-containing complexes function as co-activators and are involved in transcription factor and global histone acetylation. Oncogene 26: 5341–5357, 2007 17694077 10.1038/sj.onc.1210604

[56] Nogueiras R Habegger KM Chaudhary N Finan B Banks AS Dietrich MO Horvath TL Sinclair DA Pfluger PT Tschop MH. Sirtuin 1 and sirtuin 3: physiological modulators of metabolism. Physiol Rev 92: 1479–1514, 2012 22811431 10.1152/physrev.00022.2011 PMC3746174

[57] Norvell A McMahon SB. Cell biology. Rise of the rival. Science 327: 964–965, 2010 20167774 10.1126/science.1187159

[58] Philp A Chen A Lan D Meyer GA Murphy AN Knapp AE Olfert IM McCurdy CE Marcotte GR Hogan MC Baar K Schenk S. Sirtuin 1 (SIRT1) deacetylase activity is not required for mitochondrial biogenesis or peroxisome proliferator-activated receptor-γ coactivator-1α (PGC-1α) deacetylation following endurance exercise. J Biol Chem 286: 30561–30570, 2011 21757760 10.1074/jbc.M111.261685 PMC3162416

[59] Philp A Schenk S. Unraveling the complexities of SIRT1-mediated mitochondrial regulation in skeletal muscle. Exerc Sport Sci Rev 41: 174–181, 2013 23792490 10.1097/JES.0b013e3182956803 PMC3707796

[60] Potthoff MJ Wu H Arnold MA Shelton JM Backs J McAnally J Richardson JA Bassel-Duby R Olson EN. Histone deacetylase degradation and MEF2 activation promote the formation of slow-twitch myofibers. J Clin Invest 117: 2459–2467, 2007 17786239 10.1172/JCI31960 PMC1957540

[61] Puigserver P Adelmant G Wu Z Fan M Xu J O'Malley B Spiegelman BM. Activation of PPARγ coactivator-1 through transcription factor docking. Science 286: 1368–1371, 1999 10558993 10.1126/science.286.5443.1368

[62] Roth SY Denu JM Allis CD. Histone acetyltransferases. Annu Rev Biochem 70: 81–120, 2001 11395403 10.1146/annurev.biochem.70.1.81

[63] Ruiz-Carrillo A Wangh LJ Allfrey VG. Processing of newly synthesized histone molecules. Science 190: 117–128, 1975 1166303 10.1126/science.1166303

[64] Sartorelli V Huang J Hamamori Y Kedes L. Molecular mechanisms of myogenic coactivation by p300: direct interaction with the activation domain of MyoD and with the MADS box of MEF2C. Mol Cell Biol 17: 1010–1026, 1997 9001254 10.1128/mcb.17.2.1010 PMC231826

[65] Scott I Webster BR Li JH Sack MN. Identification of a molecular component of the mitochondrial acetyltransferase programme: a novel role for GCN5L1. Biochem J 443: 655–661, 2012 22309213 10.1042/BJ20120118 PMC7461726

[66] Senf SM Sandesara PB Reed SA Judge AR. p300 Acetyltransferase activity differentially regulates the localization and activity of the FOXO homologues in skeletal muscle. Am J Physiol Cell Physiol 300: C1490–C1501, 2011 21389279 10.1152/ajpcell.00255.2010 PMC3118617

[67] Sol EM Wagner SA Weinert BT Kumar A Kim HS Deng CX Choudhary C. Proteomic investigations of lysine acetylation identify diverse substrates of mitochondrial deacetylase sirt3. PLos One 7: e50545, 2012 23236377 10.1371/journal.pone.0050545 PMC3517600

[68] Still AJ Floyd BJ Hebert AS Bingman CA Carson JJ Gunderson DR Dolan BK Grimsrud PA Dittenhafer-Reed KE Stapleton DS Keller MP Westphall MS Denu JM Attie AD Coon JJ Pagliarini DJ. Quantification of mitochondrial acetylation dynamics highlights prominent sites of metabolic regulation. J Biol Chem 288: 26209–26219, 2013 23864654 10.1074/jbc.M113.483396 PMC3764825

[69] Sutendra G Kinnaird A Dromparis P Paulin R Stenson TH Haromy A Hashimoto K Zhang N Flaim E Michelakis ED. A nuclear pyruvate dehydrogenase complex is important for the generation of acetyl-CoA and histone acetylation. Cell 158: 84–97, 2014 24995980 10.1016/j.cell.2014.04.046

[70] Thai MV Guruswamy S Cao KT Pessin JE Olson AL. Myocyte enhancer factor 2 (MEF2)-binding site is required for GLUT4 gene expression in transgenic mice. Regulation of MEF2 DNA binding activity in insulin-deficient diabetes. J Biol Chem 273: 14285–14292, 1998 9603935 10.1074/jbc.273.23.14285

[71] Tse C Sera T Wolffe AP Hansen JC. Disruption of higher-order folding by core histone acetylation dramatically enhances transcription of nucleosomal arrays by RNA polymerase III. Mol Cell Biol 18: 4629–4638, 1998 9671473 10.1128/mcb.18.8.4629 PMC109049

[72] Wagner GR Payne RM. Widespread and enzyme-independent *N*^ε^-acetylation and *N*^ε^-succinylation of proteins in the chemical conditions of the mitochondrial matrix. J Biol Chem 288: 29036–29045, 2013 23946487 10.1074/jbc.M113.486753 PMC3790002

[73] Wallberg AE Yamamura S Malik S Spiegelman BM Roeder RG. Coordination of p300-mediated chromatin remodeling and TRAP/mediator function through coactivator PGC-1α. Mol Cell 12: 1137–1149, 2003 14636573 10.1016/s1097-2765(03)00391-5

[74] Wang L Tang Y Cole PA Marmorstein R. Structure and chemistry of the p300/CBP and Rtt109 histone acetyltransferases: implications for histone acetyltransferase evolution and function. Curr Opin Struct Biol 18: 741–747, 2008 18845255 10.1016/j.sbi.2008.09.004 PMC2643075

[75] Wang Q Zhang Y Yang C Xiong H Lin Y Yao J Li H Xie L Zhao W Yao Y Ning ZB Zeng R Xiong Y Guan KL Zhao S Zhao GP. Acetylation of metabolic enzymes coordinates carbon source utilization and metabolic flux. Science 327: 1004–1007, 2010 20167787 10.1126/science.1179687 PMC4183141

[76] Wangh L Ruiz-Carrillo A Allfrey VG. Separation and analysis of histone subfractions differing in their degree of acetylation: some correlations with genetic activity in development. Arch Biochem Biophys 150: 44–56, 1972 5028081 10.1016/0003-9861(72)90008-2

[77] Weinert BT Iesmantavicius V Wagner SA Scholz C Gummesson B Beli P Nystrom T Choudhary C. Acetyl-phosphate is a critical determinant of lysine acetylation in E. coli. Mol Cell 51: 265–272, 2013 10.1016/j.molcel.2013.06.003 23830618

[78] Yang L Vaitheesvaran B Hartil K Robinson AJ Hoopmann MR Eng JK Kurland IJ Bruce JE. The fasted/fed mouse metabolic acetylome: *N*^6^-acetylation differences suggest acetylation coordinates organ-specific fuel switching. J Proteome Res 10: 4134–4149, 2011 21728379 10.1021/pr200313x PMC3204869

[79] Yang XJ Seto E. Lysine acetylation: codified crosstalk with other posttranslational modifications. Mol Cell 31: 449–461, 2008 18722172 10.1016/j.molcel.2008.07.002 PMC2551738

[80] Zhao S Xu W Jiang W Yu W Lin Y Zhang T Yao J Zhou L Zeng Y Li H Li Y Shi J An W Hancock SM He F Qin L Chin J Yang P Chen X Lei Q Xiong Y Guan KL. Regulation of cellular metabolism by protein lysine acetylation. Science 327: 1000–1004, 2010 20167786 10.1126/science.1179689 PMC3232675

[81] Zhao X Sternsdorf T Bolger TA Evans RM Yao TP. Regulation of MEF2 by histone deacetylase 4- and SIRT1 deacetylase-mediated lysine modifications. Mol Cell Biol 25: 8456–8464, 2005 16166628 10.1128/MCB.25.19.8456-8464.2005 PMC1265742

